# CD47 blockade reverses resistance to HDAC inhibitor by liberating anti-tumor capacity of macrophages

**DOI:** 10.1186/s13046-025-03335-5

**Published:** 2025-02-24

**Authors:** Xutao Xu, Qianqian Wang, Ke Guo, Junjie Xu, Yunkun Lu, Huijuan Chen, Weilin Hu, Yilin Fu, Lu Sun, Ying He, Zhehang Chen, Wenhao Xia, Mengtian Pan, Beibei Lin, Wenjuan Yang, Qingqing Wang, Zhenzhen Wen, Qian Cao, Peng Xiao

**Affiliations:** 1 Department of Gastroenterology, Sir Run Run Shaw Hospital, Zhejiang University School of Medicine, Hangzhou, China; 2 Department of General Surgery, Sir Run Run Shaw Hospital, Zhejiang University School of Medicine, Hangzhou, China; 3 Department of Microbiology, Zhejiang University School of Medicine, Hangzhou, China; 4 China Medical University-the Queen’S University of Belfast Joint College, Shenyang, China; 5 Department of Microbiology and Immunology, School of Medicine, University of North Carolina at Chapel Hill, Chapel Hill, NC USA; 6 The First Affiliated Hospital of Huzhou University, Huzhou, China; 7 Institute of Immunology, Zhejiang University School of Medicine, Hangzhou, China

**Keywords:** HDAC inhibitor, Macrophage, Cancer, CD47, Tumor microenvironment

## Abstract

**Background:**

Targeting oncogenic histone modification by histone deacetylase inhibitors (HDACis) demonstrates promising prospects in clinical cancer treatment, whereas a notable proportion of patients cannot benefit from HDACi therapy. This study aims to explore how HDACi influences the tumor microenvironment, in order to identify potential targets for reversing the resistance to HDACi therapies.

**Methods:**

Macrophage infiltration was compared between HDACi-responding and HDACi-nonresponding cancer patients. The impact of HDACis on the phagocytic capacity of macrophages was investigated through macrophage-tumor cell co-culture system. CD47 expression in tumor cell lines and patient-derived organoids was evaluated by quantitative polymerase chain reaction (QPCR) and flow cytometry. Mechanistic studies were conducted through co-immunoprecipitation (co-IP) and chromatin immunoprecipitation (ChIP). The synergistic effect of HDACis and CD47 neutralizing antibody was assessed in subcutaneous murine tumor models. Bioinformatics approaches were adopted to analyze how macrophage infiltration determines the prognostic significance of CD47 expression in cancer patients.

**Results:**

High macrophage infiltration is a determinant of therapeutic non-response to HDACi, cancer patients who did not respond to HDACi exhibit massive infiltration of tumor-associated macrophages (TAMs). TAM depletion reversed the resistance to HDACi therapy. Mechanistically, HDACi impaired the phagocytic capacity of macrophages against tumor cells through epigenetically upregulating CD47 expression. Reciprocally, HDACi-upregulated CD47 polarized macrophages towards a pro-tumor M2 phenotype through SIRPα ligation. In tumor-bearing mice, HDACi monotherapy only marginally delayed tumor progression, while the concurrent neutralization of CD47 exhibited potent anti-tumor effect through re-educating TAMs towards a tumoricidal phenotype. In cancer patients, CD47 was found to determine the prognostic significance of TAMs.

**Conclusions:**

Our study offers a rationale for targeting macrophage infiltration or blocking CD47 to sensitize HDACi therapies in cancer patients.

**Supplementary Information:**

The online version contains supplementary material available at 10.1186/s13046-025-03335-5.

## Background

In addition to oncogenic mutations, aberrant epigenetic modifications are key etiological factors of cancer [1]. Histone acetylation, being one of the most prevalent epigenetic modifications, is essential for maintaining an open chromatin structure that is associated with transcriptional activation. In contrast, removal of acetylation by histone deacetylases (HDACs) leads to condensation of the chromatin structure. Elevated HDAC expression or activity is observed in a panel of human cancers, and contributes to the tumorigenesis and progression of cancer [2]. Therefore, a range of HDAC inhibitors (HDACis) have been developed for cancer intervention over the past two decades, and have received clinical approval (e.g. belinostat, vorinostat) or are undergoing clinical trials (entinostat). While HDAC inhibitors exhibit efficacy in certain hematological malignancies like peripheral T-cell lymphoma and cutaneous T-cell lymphoma, their therapeutic effectiveness is constrained in solid tumors [3]. In addition, some HDACis demonstrate unsatisfactory single-agent activity [4, 5], emphasizing the necessity of innovating HDACi-based combinatorial treatment approaches.

The anti-tumor medication inevitably induces a dramatic change of the tumor microenvironment (TME). Although HDAC inhibitors exhibit potent cytotoxic effects on tumor cells in cell culture systems, their capability in suppressing growing tumors in vivo is limited. Hence, this points to the likelihood that the hostile TME hinders the therapeutic efficacy of HDACis. Macrophages, as a subset of innate immune cells, represent one of the most abundant stromal populations within the TME of most solid tumors [6]. Our group has summarized the crucial impacts of tissue macrophages on drug responses in various types of human diseases [7]. However, whether macrophages are involved in regulating the therapeutic effectiveness of HDACi in cancer remains unknown by far.

In the present study, we for the first time observed that a high infiltration of macrophages is associated with the non-response to HDACi therapy in cancer patients. HDACi administration reprogrammed tumor-associated macrophages (TAMs) through dual mechanisms that are dependent on CD47. The simultaneous blockade of CD47 signaling ensured optimal therapeutic response to HDACis. In addition, the molecular mechanisms underlying the HDACi-induced pathogenic reprogramming of macrophages were also explored in depth.

## Methods

### Cell culture

HCT116, SW480 cells were cultured in Dulbecco's Modified Eagle's Medium (DMEM) medium supplemented with 10% fetal bovine serum (Serana, Brandenburg, Germany). According to experimental design, tumor cells were treated with vorinostat (MedChemExpress, Shanghai, China), entinostat (MedChemExpress), ricolinostat (MedChemExpress), nicotinamide (MedChemExpress), SIS17 (MedChemExpress), TMP269 (MedChemExpress), and various SCFAs (SigmaAldrich, St. Louis, MO, USA).

### HCT116 CRC model

5 × 10^6^ HCT116 CRC cells were inoculated s.c. into 8–10 weeks old Balb/c nude mice. Mice were randomly allocated into experimental groups and housed in specific pathogen free (SPF) condition. From the day of tumor inoculation, mice were i. p. administered with 150 μl clodronate liposomes (FormuMax Scientific, Sunnyvale, CA, USA), 60 mg/kg VOR, or in combination. Injection was performed every two to three days. For butyrate administration, butyrate was supplemented in the drinking water at a final concentration of 150 mM throughout the experiments. For CD47 neutralization, 150 μg anti-CD47 antibody (BioXCell, Lebanon, NH, USA) was i. p. injected into tumor-bearing mice every two to three days. No criteria was used for including and excluding mice.

### MC38 CRC model

5 × 10^5^ MC38 CRC cells were inoculated s.c. into 8–10 weeks old C57BL/6 mice. Mice were randomly allocated into experimental groups and housed in SPF condition. From the day of tumor inoculation, mice were i. p. administered with 150 μl clodronate liposomes, 60 mg/kg VOR, or in combination. Injection was performed twice a week.

### Stool extracts

Mice were treated with an antibiotic cocktail containing 1 g/L ampicillin (Sigma-Aldrich), 1 g/L neomycin (Sigma-Aldrich), 1 g/L metronidazole (Sigma-Aldrich), and 0.5 g/L vancomycin (Sigma-Aldrich) supplemented in the drinking water for four weeks. Control mice were left untreated with antibiotics. Stools were collected, weighed, and homogenized in DMEM medium (1 ml medium per 100 mg stool). The stool solutions were centrifuged at 12,000 g for 5 min, the supernatants were collected and filtered through 0.22 μm membrane filters. The products are stool extracts that were added to the organoid culture at a 50% (v/v) ratio.

### E0771 breast cancer model

2 × 10^6^ E0771 breast cancer cells were inoculated s.c. into 8–10 weeks old, female C57BL/6 mice. Mice were randomly allocated into experimental groups and housed in SPF condition. From the day of tumor inoculation, mice were i. p. administered with 150 μl clodronate liposomes, 15 mg/kg chidamide, or in combination. Injection was performed twice a week.

### Clinical samples

Feces and tumor tissues from CRC patients were collected at the First Affiliated Hospital of Huzhou University and Sir Run Run Shaw Hospital of Zhejiang University. SCFA metabolomics was performed as we previously described [8]. For the chidamide cohort, tumor tissues were collected from patients with HR-positive, HER2-negative advanced breast cancer, who were administered with chidamide in combination with exemestane tablets. Samples were harvested before treatment. Patients with decreased or stable metastasis were defined as responders, patients with progressive metastasis were defined as non-responders. Experiments using clinical specimens were conducted under the approval from the Medical Ethics Committee of Sir Run Run Shaw Hospital of Zhejiang University (20,220,103–56), and the First Affiliated Hospital of Huzhou University (2021KYLL-Y-005). Informed consents were obtained from all patients.

### Macrophage phagocytosis assay

Murine peritoneal macrophages or human PBMC-derived macrophages were obtained and cultured as we previously described [9]. Tumor cells were cultured in a 6-well plate, stimulated with 0.5 μM VOR or 1 mM butyrate for 24 h, washed twice with PBS, and digested with trypsin. Cell suspensions were labeled with 5 μM CFSE at 37℃ for 10 min, washed with PBS, and seeded into 12-well plates. After cell adhesion, macrophages were added to the 12-well plates to co-culture with tumor cells overnight. On the next day, the co-cultured cells were stained with APC-F4/80 (for mouse macrophages) or APC-CD11b (for human macrophages) (Biolegend, San Diego, CA, USA), the phagocytosis of tumor cells by macrophages was evaluated by flow cytometry.

### QPCR

QPCR was performed as we previously described [9]. The primer sequences are: CD47 forward: AGAAGGTGAAACGATCATCGAGC; CD47 reverse: CTCATCCATACCACCGGATCT. CD206 forward: TCCGGGTGCTGTTCTCCTA; CD206 reverse: CCAGTCTGTTTTTGATGGCACT. β-actin forward: CATGTACGTTGCTATCCAGGC; β-actin reverse: CTCCTTAATGTCACGCACGAT. The primer sequences used for the screening of macrophage-related immune checkpoint molecules are available from the corresponding author upon request.

### Organoid culture

The isolation and culture of organoids from CRC patients were performed using human IntestiCult™ Organoid Growth Medium (STEMCELL, Cambridge, MA, USA) as we previously described [8].

### Co-immunoprecipitation and Western blot

Butyrate- (1 mM, 6 h) or PBS-treated HCT116 cells were lysed using immunoprecipitation lysis buffer (Beyotime) on ice for 30 min, the supernatants were obtained by centrifugation. Protein A magnetic beads was incubated with primary anti-Sp1 antibody (Cell Signaling Technology, Danvers, MA, USA) by rotating 20 min at room temperature. After washing with PBS-T, the antibody-binded beads were incubated with the cell lysates at 4 °C overnight. Then the beads were washed and eluted using immunoprecipitation lysis buffer. The eluents containing interacted proteins were separated by sodium dodecyl sulphate–polyacrylamide gel electrophoresis (SDS-PAGE), followed by Western blot analysis using anti-Sp1 antibody (Cell Signaling Technology) and anti-HDAC1 antibody (Cell Signaling Technology). For the detection of histone H3 acetylation, anti-acetyl-histone H3-Lys18 antibody (Cell Signaling Technology) and anti-histone H3 antibody (Cell Signaling Technology) were used.

### ChIP assay

ChIP assay was performed using SimpleCHIP Enzymatic Chromatin IP Kit (Cell Signaling Technology). According to the manufacturer’s protocol, cells were crosslinked by 1% formaldehyde, neutralized with 125 mM glycine, and then harvested with ice-cold PBS containing protease inhibitor cocktail. Cell pellets were digested by micrococcal nuclease at 37 °C for 20 min, followed by sonication for 3 sets of 20 s pulses to obtain appropriate chromatin lysates. The lysates were clarified by centrifugation and then the supernatants were incubated with anti-Sp1 rabbit mAb (Cell Signaling Technology) at 4 °C overnight. Thereafter, protein G magnetic beads were added and a two-hour incubation with rotation was conducted. After chromatin elution and DNA purification, DNA products were subjected to QPCR analysis for quantification.

### Flow cytometry

HCT116 tumors were digested into single cell suspensions using 300 U/mL collagenase type IV (Worthington, Lakewood, NJ, USA). Single cell suspensions were filtered through 70 μm cell strainers and incubated with TruStain FcX™ PLUS (Biolegend) on ice for 10 min. Cells were stained with anti-F4/80 (Biolegend), anti-CD206 (Biolegend), and Zombie Violet™ Viability dye (Biolegend) on ice for 20 min. For the staining of CD47, trypsinized tumor cells were incubated with anti-CD47 antibody (Biolegend) on ice for 20 min. After PBS washing, cells were subjected to flow cytometry on a NovoCyteTM Flow Cytometer (Agilent, Santa Clara, CA, USA).

### Statistical analysis

Data were presented as mean ± standard deviation (SD). Statistical analysis was conducted using GraphPad Prism 8 software (Boston, MA, USA). Unpaired two-tailed Student’s t-test, paired two-tailed Student’s t-test, log-rank test, and Spearman’s rank correlation test were used where appropriate. P values less than 0.05 were considered significant.

## Results

### Tumors with high macrophage infiltration exhibit resistance to HDACi therapy

**Fig. 1:**
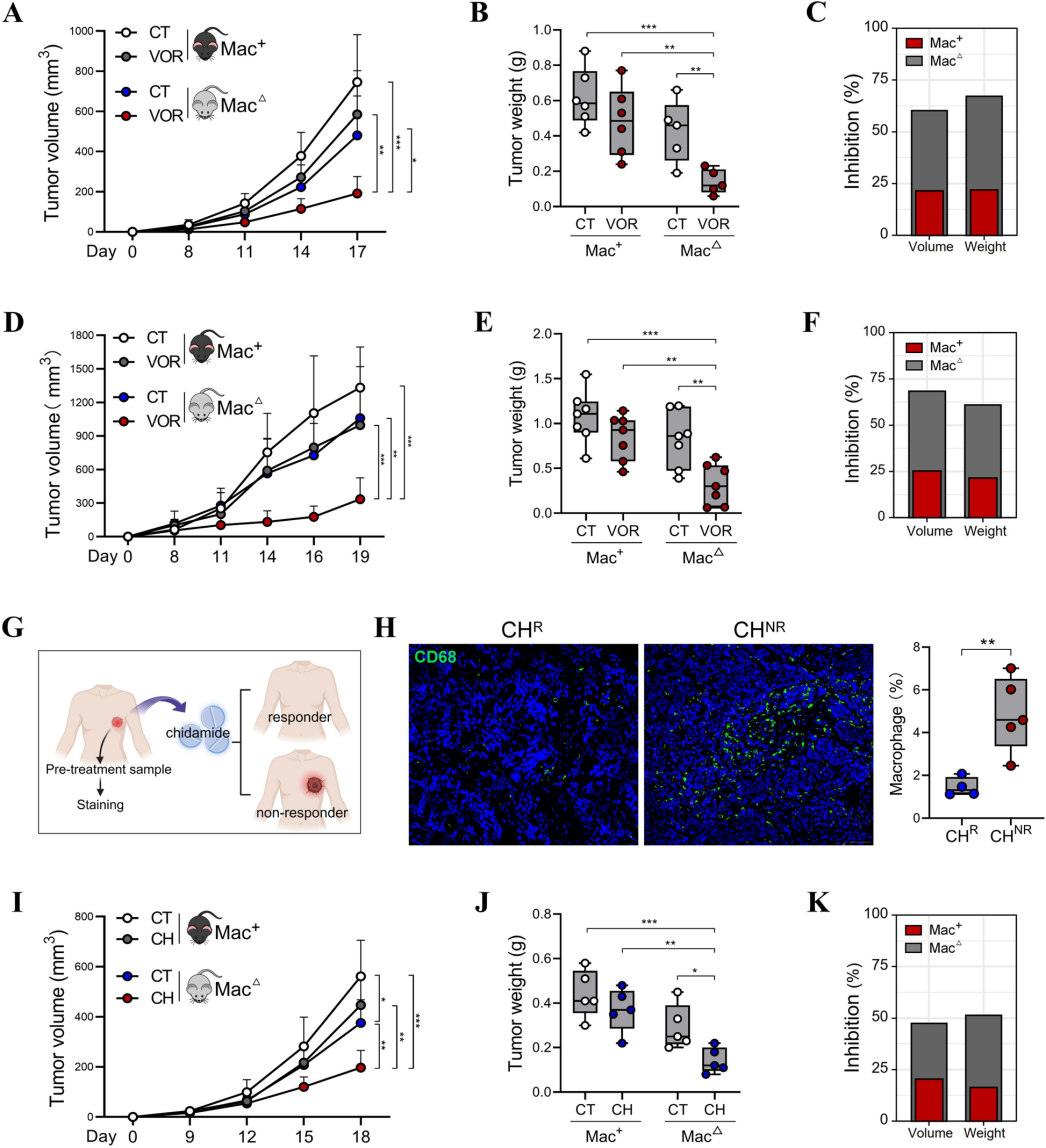
Macrophages determine the therapeutic outcome of HDACi therapy. **A-C** MC38 CRC cells were subcutaneously (s.c.) inoculated into control (Mac^+^) or clodronate liposome-treated (macrophage-depleted, Mac^△^) C57BL/6 mice (*n* = 5–6/group), which were intraperitoneally (i.p.) administered with 60 mg/kg VOR. Tumor growth was measured (**A**); tumor weight was evaluated at experimental endpoint **B**; the capability of VOR in suppressing tumor volume and weight in Mac^+^ mice or Mac^△^ mice were calculated **C**. **D**-**F** HCT116 CRC cells were s.c. inoculated into Mac^+^ or Mac^△^ nude mice (*n* = 7/group), which were i.p. administered with 60 mg/kg VOR. Tumor growth was measured **D**; tumor weight was evaluated at experimental endpoint **E**; the capability of VOR in suppressing tumor volume and weight in Mac^+^ mice or Mac^△^ mice were calculated **F**. **G**, **H** Tumor tissues from chidamide (CH)-treated breast cancer patients were collected before treatment, and stained with CD68 (G, created with http://BioRender.com), the percentages of CD68^+^ macrophages were compared between CH^R^ and CH^NR^ patients (H). (I-K) E0771 breast cancer cells were s.c. inoculated into Mac^+^ or Mac^△^ C57BL/6 mice (*n* = 5/group), which were i.p. administered with 15 mg/kg chidamide. Tumor growth was measured **I**, tumor weight was evaluated at experimental endpoint **J**; the capability of chidamide in suppressing tumor volume and weight in Mac^+^ mice or Mac^△^ mice were calculated (**K**). *p < 0.05; **p < 0.01; ***p < 0.001, unpaired, two-tailed Student’s t test

Latest clinical trials have demonstrated the encouraging efficacy of HDACi therapy in solid tumors, such as colorectal cancer (CRC) [10, 11]. In an effort to explore the potential involvement of macrophages in HDACi resistance, we employed a murine MC38 CRC model, during which macrophages were eliminated using clodronate liposomes. The macrophage intact (Mac^+^) or macrophage-depleted mice (Mac^△^) were then administered with vorinostat (VOR). Of note, although VOR only slightly delayed tumor growth and reduced tumor weight in Mac^+^ mice, its therapeutic effectiveness was remarkably amplified in Mac^△^ mice (Fig. 1A-C). Furthermore, we inoculated human CRC cell line HCT116 into athymic nude mice, in which T cell responses are lacking. Similar to the MC38 model, VOR exhibited limited effectiveness in suppressing tumor growth in Mac^+^ nude mice, while the depletion of macrophages drastically sensitized the anti-tumor efficacy of VOR (Fig. 1D-F). These findings suggest that macrophages compromise the therapeutic efficacy of VOR, and the macrophage-mediated HDACi resistance is relatively independent of adaptive immune responses.

The preclinical evidence from murine models prompted us to further explore the clinical relevance between macrophage infiltration and the therapeutic outcomes of HDACi. For this purpose, we collected tumor tissues from breast cancer patients who underwent administration of chidamide, a HDACi approved by Chinese Food and Drug Administration (CFDA) for the treatment of peripheral T-cell lymphoma and advanced breast cancer. Samples were harvested prior to the initiation of chidamide treatment. Based on their prognosis, patients were categorized into chidamide responder group (CH^R^) and chidamide non-responder group (CH^NR^). Strikingly, CH^NR^ tumors exhibited a massive infiltration of macrophages, which was not observed in the CH^R^ group (Fig. 1G, H), suggesting that macrophage abundance is potentially useful for the pre-identification of patient response.

In order to investigate the possible causative role of macrophages in chidamide resistance, an E0771 breast cancer model was established. Consistent with the clinical association between high macrophage percentage and chidamide nonresponse, chidamide administration strongly suppressed tumor growth and decreased tumor weight in Mac^△^ mice but not in Mac^+^ mice (Fig. 1I-K). Therefore, we for the first time unveiled the primary role of macrophages in the ineffectiveness of HDACi therapy.

### VOR counteracts macrophage-mediated tumor phagocytosis through inducing CD47 expression

**Fig. 2:**
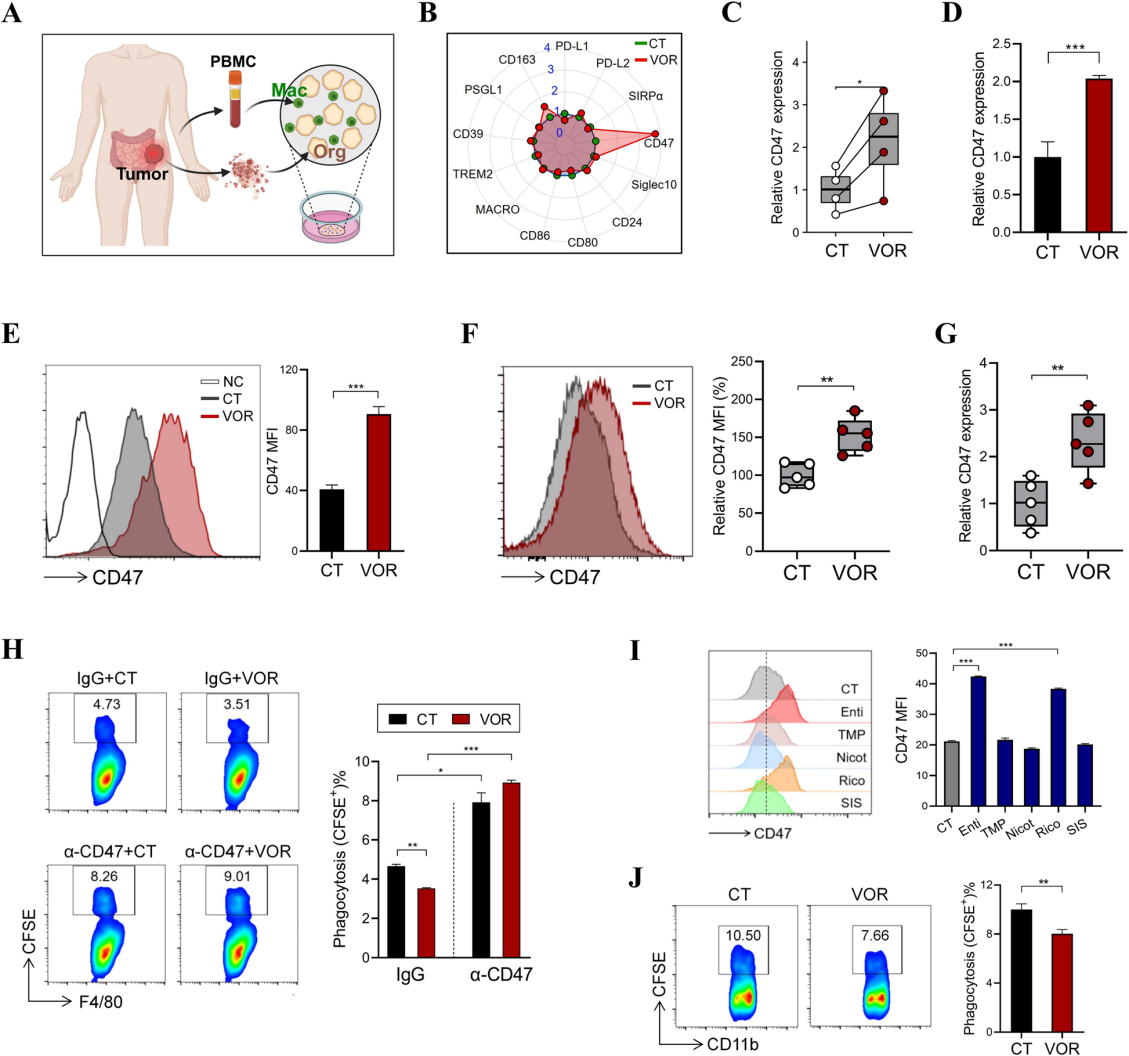
VOR suppresses the phagocytosis of macrophages against CRC cells through CD47 induction. **A** Scheme illustrating the co-culture of CRC patient organoids with human primary macrophages (Created with http://BioRender.com). **B** The organoid-macrophage co-culture was treated with 0.5 μM VOR for 12 h, the expression of indicated genes was evaluated by QPCR. Blue fonts indicate fold change relative to control cells. **C** CRC patient organoids were stimulated with 0.5 μM VOR for 12 h, the expression of CD47 was evaluated by QPCR. **D**, **E** HCT116 cells were stimulated with VOR for 12 h, the expression of CD47 was evaluated by QPCR (**D**) and flow cytometry. NC = negative control, CT = control **E**. **F**, **G** Nude mice were s.c. inoculated with HCT116 cells, followed by the administration of VOR for 19 days (*n* = 5/group). The surface levels of CD47 in live tumor cells were evaluated by flow cytometry (F); The expression of CD47 in tumor tissues was evaluated by QPCR **G**. **H** HCT116 cells were treated with VOR for 24 h and labeled with Carboxyfluorescein succinimidyl ester (CFSE), followed by the co-incubation with mouse peritoneal macrophages overnight in the presence of IgG1 or α-CD47 (50 μg/mL). The phagocytosis of macrophages was evaluated by flow cytometry. **I** HCT116 cells were treated with the indicated HDAC subtype inhibitors (0.5 μM) for 24 h, the surface levels of CD47 were evaluated by flow cytometry. **J** HCT116 cells were treated with 0.5 μM VOR for 24 h and labeled with CFSE, followed by the co-incubation with human PBMC-derived primary macrophages overnight. The phagocytosis of macrophages was evaluated by flow cytometry. *p < 0.05; **p < 0.01; ***p < 0.001, unpaired, two-tailed Student’s t test; or paired, two-tailed Student’s t test for Fig. 2C

Based on the above findings, we speculated that HDAC inhibition leads to the functional reprogramming of TAMs, either directly or indirectly through regulating tumor-macrophage crosstalk. Therefore, we established a co-culture system, in which tumor organoids from CRC patients were cultured with primary human macrophages (Fig. 2A). The co-cultured cells were exposed to VOR, then the levels of a panel of macrophage-related immune checkpoint molecules [12] were screened. Among these markers, we found a notable increase in the expression of CD47 (Fig. 2B), a surface protein which prevents the phagocytic elimination by macrophages when expressed on malignant cells. The induction of CD47 upon VOR challenge was further validated using non-cocultured CRC organoids and HCT116 CRC cells by QPCR and flow cytometry (Fig. 2C-E). Congruent with the in vitro results, VOR administration significantly boosted the levels of CD47 in the tumor tissues of HCT116-bearing mice (Fig. 2F, G).

Given the higher expression of CD47 on VOR-exposed cells, we wondered whether VOR can impair the phagocytic elimination of CRC cells. Indeed, flow cytometry results demonstrated that VOR significantly diminished the phagocytic capacity of macrophages against co-cultured HCT116 cells, a phenomenon that was reversed by a CD47 blocking antibody (α-CD47) (Fig. 2H). We also tested the effect of VOR on CD47 expression in SW480 CRC cells, PANC-1 pancreatic cancer cells and MDA-MB-231 breast cancer cells. Similar to HCT116 cells, VOR treatment significantly increased the mRNA and protein levels of CD47 (Fig. S1, S2), and impaired the phagocytosis of macrophages against SW480, PANC-1 and MDA-MB-231 cells (Fig. S3).

VOR functions as a pan HDAC inhibitor. To assess the capability of each HDAC subtype in upregulating CD47 expression, we treated CRC cells with inhibitors for type I HDAC (entinostat), type IIa HDAC (TMP269), type IIb HDAC (ricolinostat), type III HDAC (nicotinamide), or type IV HDAC (SIS17). The results showed that inhibition of type I HDAC (HDAC1/2/3) and type IIb HDAC (HDAC6) caused a significant upregulation of CD47, whereas inhibition of other HDAC subtypes did not exhibit obvious effect on CD47 (Fig. 2I). We further used human peripheral blood mononuclear cell (PBMC)-derived primary macrophages, and observed significantly lowered phagocytic capacity of macrophages against VOR-primed CRC cells compared with control CRC cells, recapitulating the results from murine macrophages (Fig. 2J). Therefore, HDAC inhibition strengthened the “don’t eat me” signal through upregulating CD47 expression on tumor cells.

### Endogenous HDACi connects microbial signals and CD47 expression in CRC tumor microenvironment

**Fig. 3:**
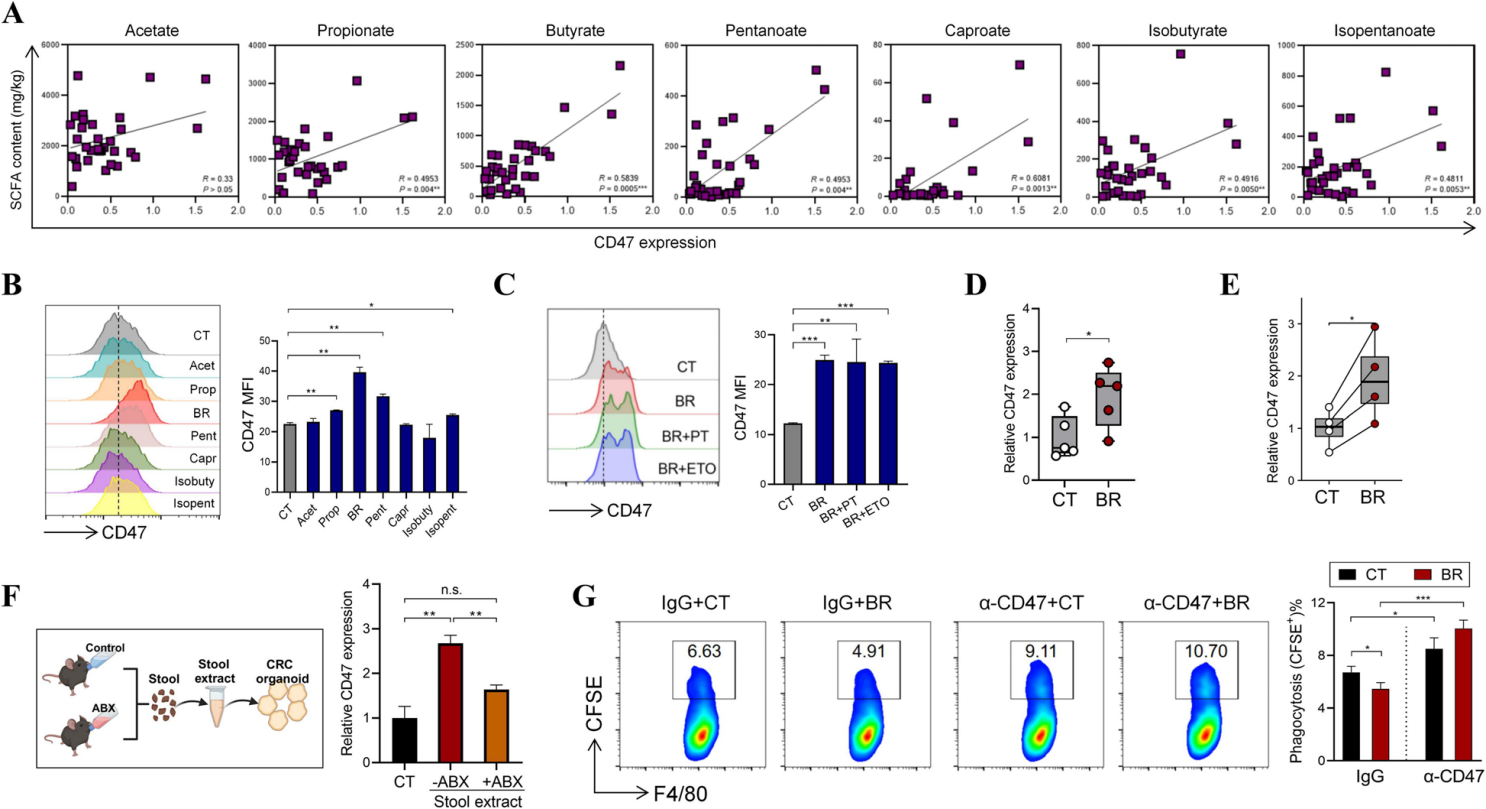
Butyrate promote CD47-mediated phagocytic inhibition. **A** Fecal samples were collected from CRC patients. The concentrations of various SCFAs were measured by metabolomics in our previous study, the expression of CD47 in matched tumor tissues was evaluated by QPCR. The correlations between tumor CD47 expression and fecal SCFA levels were analyzed using Spearman’s correlation test. (*n* = 32. For isobutyrate or caproate, *n* = 31 or 25 respectively, as some patients have low levels of isobutyrate or caproate which were below the detection threshold). **B** HCT116 cells were stimulated with 2 mM various SCFAs for 24 h, the expression of CD47 was evaluated by flow cytometry. BR = butyrate. **C** HCT116 cells were pretreated with pertussis toxin (PT) or etomoxir (ETO) for 2 h, followed by butyrate stimulation for 24 h, CD47 expression was measured by flow cytometry. **D** Nude mice were s.c. inoculated with HCT116 cells, followed by the administration of 150 mM butyrate in drinking water for 18 days (*n* = 5/group). The expression of CD47 in tumor tissues was evaluated by QPCR. **E** Organoids from CRC patients were stimulated with 1 mM butyrate for 12 h, the expression of CD47 was evaluated by QPCR. **F** Mice were fed with antibiotic cocktails for four weeks. Stools were collected to prepare stool extracts, which was used to treat CRC organoids (left, created with http://BioRender.com). The expression of CD47 in organoids 12 h after stimulation was measured by QPCR (right). **G** HCT116 cells were treated with 1 mM BR for 24 h and labeled with CFSE, followed by the co-incubation with mouse peritoneal macrophages overnight in the presence of IgG1 or α-CD47 (50 μg/mL). The phagocytosis of macrophages was evaluated by flow cytometry. *p < 0.05; **p < 0.01; ***p < 0.001, unpaired, two-tailed Student’s t test; or paired, two-tailed Student’s t test for Fig. 3E

Apart from synthetic HDACi drugs, short-chain fatty acids (SCFAs), particularly butyrate, act as endogenous HDACi produced by microbial fermentation in the gut. Through performing metabolomics, we have previously reported that the levels of multiple SCFAs were decreased in CRC patients compared to healthy individuals [8]. In the current study, we noticed that there exist positive correlations between CD47 expression and fecal concentrations of SCFAs in CRC patients, with butyrate showing the most significant correlation (Fig. 3A). When we exposed CRC cells to these SCFAs respectively, it was found that butyrate robustly upregulated CD47 expression. Propionate, pentanoate, and isopentanoate also enhanced CD47 expression, albeit to a lesser extent compared to butyrate (Fig. 3B). The CD47-inducing effect of butyrate was not compromised by pertussis toxin (a GPCR inhibitor) or etomoxir (a CPT1 inhibitor), ruling out the possibility that butyrate stimulates CD47 expression by activating GPCR signaling or through the metabolic pathway (Fig. 3C). In HCT116-bearing mice, butyrate administration led to significantly higher CD47 expression in the tumor tissues (Fig. 3D).

In line with cell line results, butyrate treatment significantly upregulated CD47 expression in organoids from CRC patients (Fig. 3E). Butyrate is primarily produced by microbial fermentation in the large intestine. For the purpose of verifying the role of gut microbiota on CD47 induction, we exposed CRC patient organoids to stool extracts derived from control mice or mice fed with antibiotic cocktails. While stool extracts from control mice remarkably enhanced CD47 expression in organoids, those from antibiotic-treated mice exhibited substantially weaker ability in CD47 induction (Fig. 3F), indicating that microbial products play a major role in supporting CD47 expression in CRC cells. Consistent with its CD47-inducing property, butyrate treatment significantly prevented CRC cells from being phagocytosed by macrophages, whereas this effect was counteracted by CD47 blocking antibody (Fig. 3G). Therefore, we identify butyrate as a novel mediator that bridges microbial signals and CD47 expression in CRC tumor microenvironment.

### HDACi-induced CD47 triggers an intrinsic signaling in macrophages to potentiate M2 polarization

**Fig. 4:**
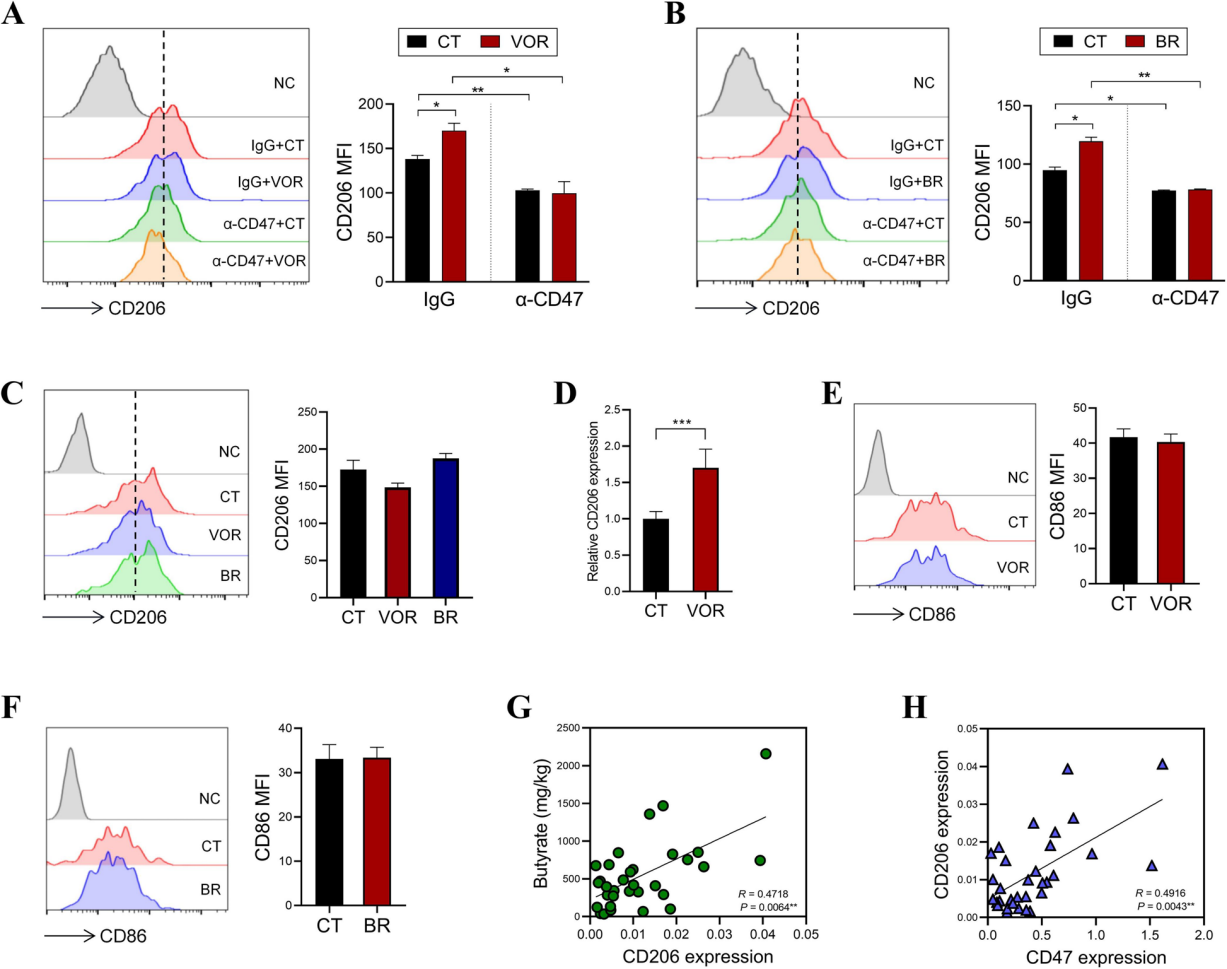
HDACis facilitate M2 polarization through promoting SIRPα ligation of CD47. (A, B) HCT116 cells were treated with 0.5 μM VOR (**A**) or 1 mM BR (**B**) for 24 h, followed by co-incubation with mouse peritoneal macrophages overnight in the presence of IgG1 or α-CD47. The expression of CD206 on F4/80^+^ macrophages was evaluated by flow cytometry. **C** HCT116 cells were treated with 0.5 μM VOR or 1 mM BR for 24 h, followed by co-incubation with mouse peritoneal macrophages overnight in the presence of 50 μg/mL SIRPα blocking antibody (α-SIRPα). The expression of CD206 on F4/80^+^ macrophages was evaluated by flow cytometry. **D** The organoid-macrophage co-culture system was established and treated as in Fig. 2A, the expression of CD206 was evaluated by QPCR. **E**, **F** HCT116 cells were treated with 0.5 μM VOR (C) or 1 mM BR (**D**) for 24 h, followed by co-incubation with mouse peritoneal macrophages overnight. The expression of CD86 on F4/80^+^ macrophages was evaluated by flow cytometry. **G** The correlation between tumor CD206 expression and fecal butyrate levels in CRC patients was analyzed using Spearman’s correlation test. **H** The correlation between the expression of CD206 and CD47 in tumor tissues from CRC patients was analyzed using Spearman’s correlation test

In addition to providing a "don’t eat me" signal, the engagement of CD47 with SIRPα induces tyrosine phosphorylation of the cytoplasmic domain of SIRPα [13]. The CD47-dependent SIRPα intrinsic signaling has been documented to regulate the functional homeostasis of dendritic cells [14, 15]. To examine the impact of HDACi-stimulated CD47 on the pro-tumor characteristics of macrophages, we co-cultured macrophages with HDACi-challenged CRC cells. The results showed that macrophages co-cultured with VOR-primed or BR-primed CRC cells expressed significantly higher levels of CD206 in comparison to those co-cultured with control CRC cells (Fig. 4A, B), indicating that HDACi induces an intercellular communication that enhances the polarization of pro-tumor M2-like macrophages. Importantly, HDACi-primed CRC cells could not enhance CD206 expression on co-cultured macrophages in the presence of CD47 blocking antibody or SIRPα blocking antibody, suggesting that this effect is dependent on CD47/SIRPα axis (Fig. 4A-C). The HDACi-induced CD206 expression was further confirmed in the organoid-macrophage co-culture system (Fig. 4D). These findings indicate that the CD47 ligation of SIRPα not only prevents the phagocytosis of tumor cells by macrophages, but also triggers a reverse, intrinsic signaling in macrophages that facilitates their M2 polarization. In contrast, co-culture with HDACi-primed HCT116 cells did not obviously affect the levels of CD86, a marker for M1-like macrophages (Fig. 4E, F).

In support of the above discoveries, we observed a significant positive correlation between fecal butyrate concentrations and CD206 expression in the tumor tissues of CRC patients (Fig. 4G). Additionally, there was also a significant positive correlation between CD206 expression and CD47 expression (Fig. 4H). Hence, we uncover a novel mechanism by which HDACis amplify the M2-polariztion of macrophages through intercellular crosstalk.

### Interrupting HDAC1-Sp1 interaction is required for the HDACi-induced CD47 upregulation

**Fig. 5:**
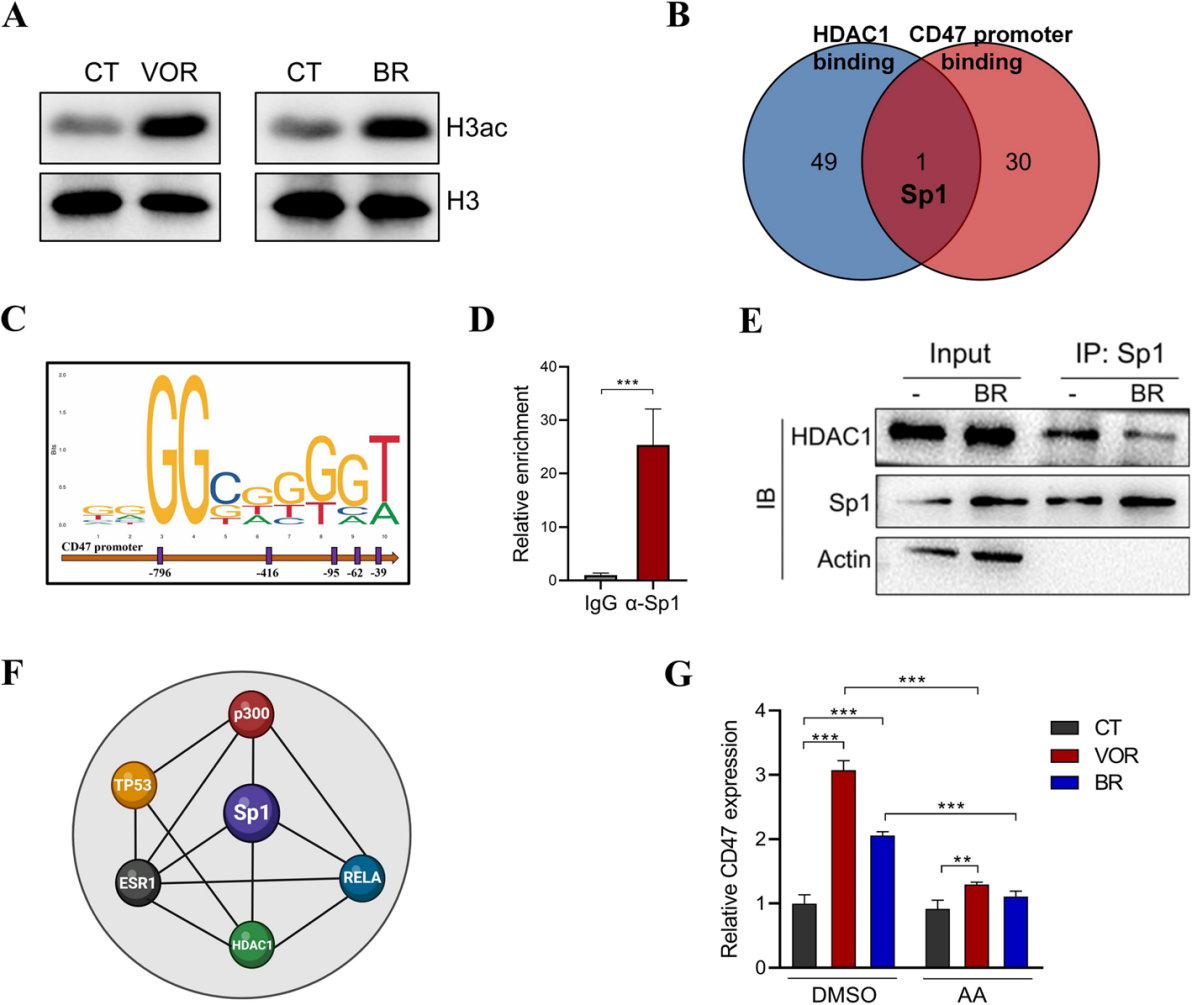
HDAC inhibition induces CD47 expression by preventing the binding of HDAC1 to Sp1. **A** HCT116 cells were treated with 0.5 μM VOR or 1 mM BR for 4 h, the acetylation of H3 histone was evaluated by Western blot. **B** Venn diagram showing proteins that can potentially interact with both CD47 promoter and HDAC1. **C** Sp1 binding sites within the CD47 promoter were predicted using the Jaspar database. **D** Sp1 binding to the CD47 promoter was evaluated by ChIP assay. **E** HCT116 cells were treated with 1 mM butyrate for 6 h, the interaction between Sp1 and HDAC1 was evaluated by Co-IP. **F** The interaction among Sp1, HDAC1, and p300 was predicted using STRING database (Created with http://BioRender.com). **G** HCT116 cells were treated with 0.5 μM VOR or 1 mM BR in the presence or absence of 20 μM AA for 12 h, the expression of CD47 was evaluated by QPCR. **p < 0.01; ***p < 0.001, unpaired, two-tailed Student’s t test

We next wondered the molecular basis of how HDACi induces CD47 expression. As anticipated, VOR or BR treatment drastically promoted the acetylation of histone H3 in CRC cells (Fig. 5A). However, HDACs lack the ability to directly bind to DNA [16], their functionality is dependent on interacting with co-regulators located at the promoters of specific genes. It has been demonstrated that the inhibition of type I HDACs led to the most robust increase in CD47 expression (Fig. 2I). Therefore, we screened the top 50 proteins capable of interacting with HDAC1 using STRING database. Among these HDAC1-binding proteins, only one (Sp1) was predicted to bind to the promoter region of CD47 gene following conducting sequence analysis using UCSC/JASPAR database (Fig. 5B). The Sp1-binding sites within the promoter region of CD47 gene were shown in Fig. 5C. The occupancy of Sp1 at CD47 promoter was further validated by a chromatin immunoprecipitation (ChIP) assay (Fig. 5D). In addition, the interaction between HDAC1 and Sp1 in CRC cells was also confirmed by co-immunoprecipitation (Co-IP). Importantly, this HDAC1-Sp1 interaction was suppressed by HDACi treatment (Fig. 5E).

The dissociation of HDAC1 from Sp1 by HDAC inhibition is known to activate the function of p300, a histone acetyltransferase (HAT) that enhances gene transcription through increasing chromatin accessibility [17]. Indeed, it is predicted that p300 can also interact with Sp1 according to the STRING database (Fig. 5F). Therefore, we treated HCT116 cells with HDACis in the presence of anacardic acid (AA), a p300 inhibitor. Although VOR and BR significantly increased CD47 expression, this effect was substantially diminished by p300 inhibition. The basal expression of CD47 was not obviously affected by p300 inhibitor (Fig. 5G). Thus, HDACis facilitate the p300-dependent transcriptional activation of CD47 through disrupting the HDAC1-Sp1 interaction.

### CD47 blockade overcomes resistance to HDAC inhibitors

**Fig. 6:**
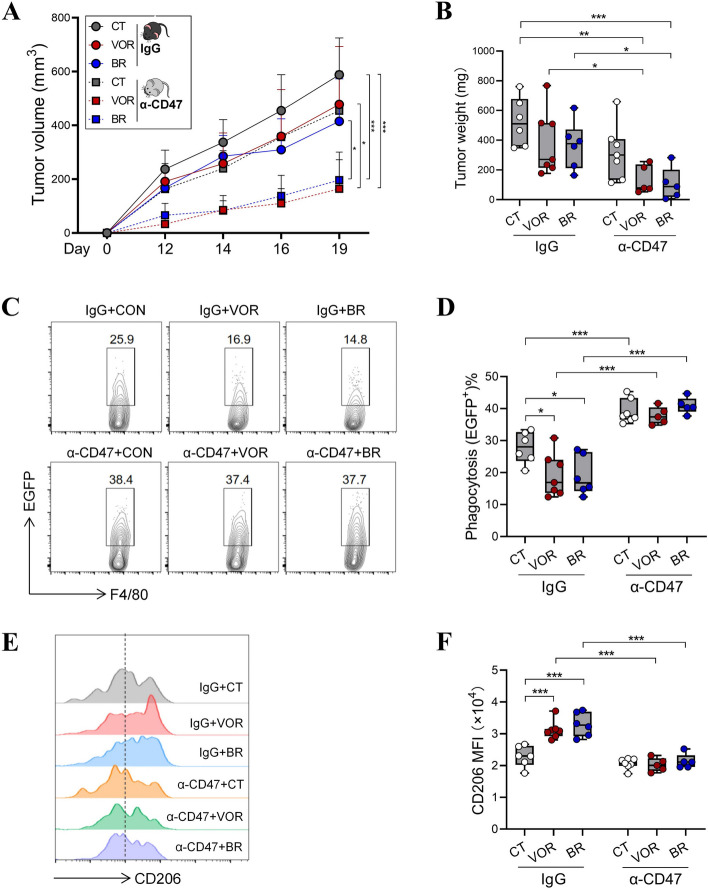
CD47 blockade sensitizes HDACi therapy by re-educating TAMs. Nude mice were s.c. inoculated with HCT116-EGFP cells, and administered with VOR (60 mg/kg) or BR (150 mM) alone or in combination with α-CD47 (*n* = 5–7/group). **A** The growth of tumor in each group was monitored. **B** Tumor weight was measured at the endpoint. **C**, **D** The phagocytosis of HCT116-EGFP cells by F4/80^+^ macrophages in tumor tissues was analyzed by flow cytometry. Representative plots (**C**) and statistical results (**D**) were shown. **E**, **F** The expression of CD206 on tumor-infiltrating F4/80^+^ macrophages was analyzed by flow cytometry. Representative plots (**E**) and statistical results (**F**) were shown. *p < 0.05; **p < 0.01; ***p < 0.001, unpaired, two-tailed Student’s t test

To explore whether CD47 blockade potentiates the therapeutic efficacy of HDACis, we inoculated mice with HCT116-EGFP cells, followed by the administration of VOR or butyrate alone, or in combination with α-CD47. As illustrated in Fig. 6A, VOR or butyrate moderately suppressed tumor development, while their anti-tumor effect was dramatically augmented by the combinatorial treatment with α-CD47 (Fig. 6A). The tumor mass exhibited a similar trend to the volume of xenograft tumors among each group (Fig. 6B), demonstrating that the simultaneous neutralization of CD47 is required for yielding the optimal therapeutic outcome of HDACis.

More importantly, both VOR and BR significantly suppressed TAM-mediated phagocytosis of HCT116-EGFP cells, while α-CD47 significantly reversed this effect (Fig. 6C, D). On the other hand, HDAC inhibition led to a significant increase in the expression of CD206 on TAMs, which was counteracted by α-CD47 (Fig. 6E, F). Therefore, CD47 blockade is essential for liberating the anti-tumor capacity of macrophages upon HDACi therapies.

### The prognostic significance of TAMs in CRC patients depends on CD47 expression

**Fig. 7:**
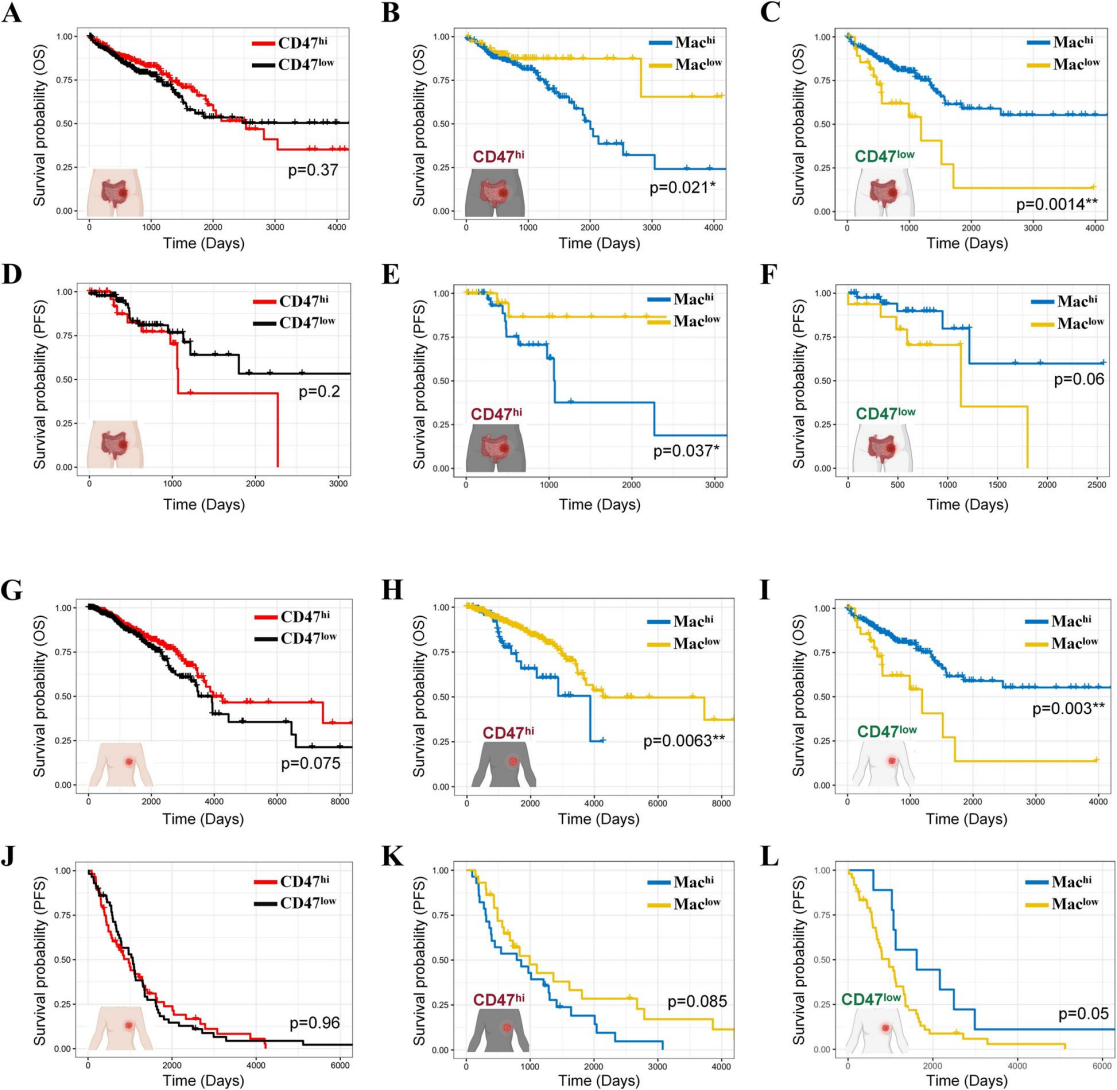
CD47 determines the prognostic significance of TAMs in cancer patients. **A**, **D** The OS (**A**) or PFS (**D**) of CRC patients were analyzed in TCGA colon adenocarcinoma (COAD)/rectum adenocarcinoma (READ) cohort based on CD47 expression. **B**, **C**, **E**, **F** The correlation between macrophage infiltration and the OS (B, C) or PFS (E, F) in CRC patients were analyzed in CD47^hi^ (**B**, **E**) and CD47^low^ (**C**, **F**) subgroups respectively. **G**, **J** The OS (G) or PFS (J) of breast cancer patients were analyzed in TCGA BRCA cohort based on CD47 expression. **H**, **I**, **K**, **L** The correlation between macrophage infiltration and the OS (**H**, **I**) or PFS (**K**, **L**) in breast cancer patients were analyzed in CD47^hi^ (**H**, **K**) and CD47^low^ (**I**, **L**) subgroups respectively. *p < 0.05; **p < 0.01; log-rank test

Given that CD47 signaling can reprogram TAMs through dual mechanisms, we next wondered the potential correlation between CD47 expression and patient prognosis in clinical settings. In The Cancer Genome Atlas (TCGA) dataset, CD47 did not exhibit a significant correlation with overall survival (OS) in CRC patients (Fig. 7A). Therefore, we further included macrophage infiltration into analysis. Strikingly, in CRC patients with high CD47 expression (CD47^hi^), high macrophage infiltration was significantly correlated with poorer OS (Fig. 7B). In sharp contrast, high macrophage infiltration was significantly correlated with favourable OS in CD47^low^ patients (Fig. 7C). When progression-free survival (PFS) was analyzed, the opposite impact of macrophages in CD47^hi^ and CD47^low^ tumors was also observed. Patients with high macrophage infiltration exhibited shorter PFS only in CD47^hi^ subgroup (Fig. 7D-F).

More encouragingly, survival analysis of breast cancer patients recapitulated the tendency in CRC cohort. High macrophage abundance predicted significantly poorer or better survival in CD47^hi^ or CD47^low^ subgroup, respectively. Conversely, low macrophage abundance was correlated with better or poorer prognosis in CD47^hi^ or CD47^low^ subgroup, respectively (Fig. 7G-L). This contrasting impact of macrophage abundance on patient prognosis suggests that CD47-mediated signaling maintains TAMs in a pro-tumor functional status. Contrarily, a low CD47 tumor microenvironment liberates the anti-tumor capability of TAMs. Thus, CD47 induction may be considered as a detrimental “side effect” of HDACi therapy. Patients with high macrophage infiltration are more likely to benefit from CD47-SIRPα immunotherapy.

### HDACi therapy induces CD47 expression in myeloid malignancies

In Fig. 1, we provided evidence showing that macrophage abundance predicted patient outcome after HDACi therapy. For the purpose of further expand the clinical relevance of our research, we analyzed a publicly available dataset from clinical trial where entinostat plus 5-azacytidine were used to treat patients with hematological malignancies [18], which currently represent the main indications of HDACis. CD34^+^ cells from patients were isolated before and after treatment and subjected to microarray analysis. As depicted in Fig. S4, the expression of CD47 in CD34^+^ cells was significantly elevated in response to treatment. Therefore, HDAC inhibition may also strengthen the “don’t eat me” signal in malignant hematological cells, which can prevent their phagocytic elimination by macrophages [19, 20].

**Fig. 8:**
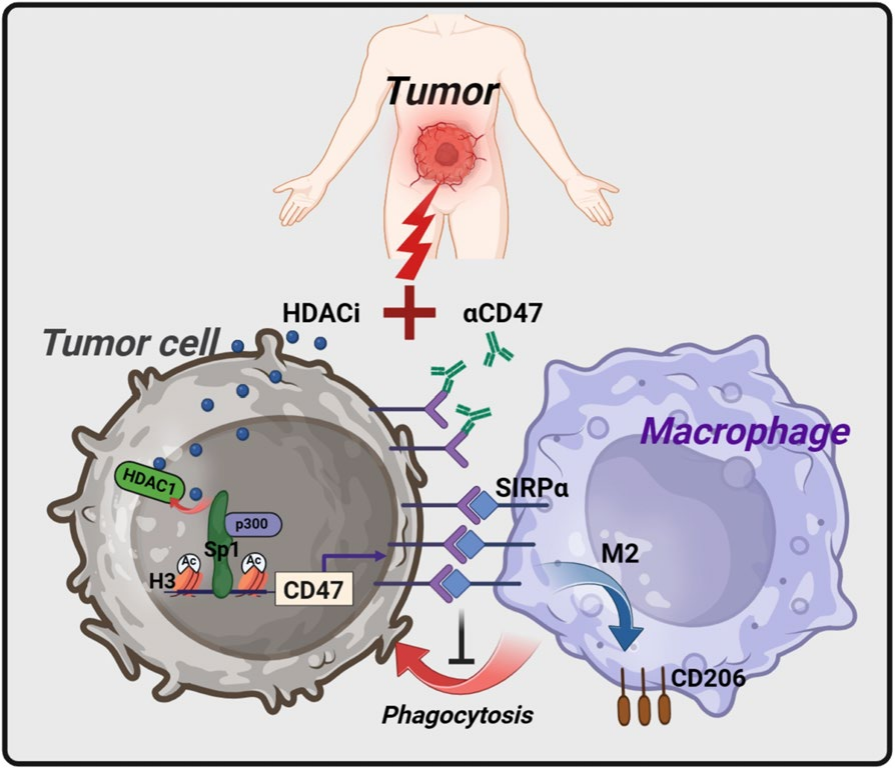
Scheme illustrating the mechanism that HDACi therapy dampens the anti-tumor macrophage responses in the TME. Blockade of CD47-SIRPα interaction overcomes the resistance to HDACi therapy. Created with http://BioRender.com

In summary, the current study emphasizes the necessity of CD47-SIRPα signaling blockade in sensitizing HDACi therapies (Fig. 8).

## Discussion

In cancer patients, the use of chemotherapy, radiotherapy, or targeted therapy drugs poses substantial survival stress to tumor cells. Regrettably, tumors frequently adapt to therapeutic interventions, either through evolving resistance to cytotoxicity or through fostering a tumor favorable microenvironment. For example, cisplatin facilitated an immunosuppressive TME by increasing PD-L1 and PD-L2 expression in ovarian cancer cells [21]. In breast cancer, doxorubicin stimulated the production of complement factors, which promoted lung metastasis through recruiting myeloid-derived suppressor cells [22]. Cetuximab, an EGFR monoclonal antibody, enhanced the functions of pro-tumor M2 macrophages [23]. In addition, the extensive tumor cell death caused by anti-tumor drugs leads to the release of various danger-associated molecular patterns (DAMPs) [24], many of which act as immunosuppressive mediators, such as HMGB1 and IL-33 [25, 26]. Therefore, the concurrent utilization of immunotherapy often causes a “double-strike” to tumors to overcome the resistance to conventional therapeutic approaches.

HDACis have gained broad application in treating cancer patients, while drug resistance occurs in a large proportion of patients. To date, how to predict and improve the therapeutic effectiveness of HDACis remains challenging. In this study, we discovered that high numbers of tumor-infiltrating macrophages is a microenvironmental characteristic of chidamide nonresponding patients compared with chidamide responders in breast cancer. Although the difference is striking between responders and non-responders, the relatively low number of samples restricted further in-depth analysis, such as establishing a receiver operating characteristic (ROC) curve. In line with the clinical observation demonstrating the potentially adverse impact of macrophages in HDACi therapy, the preclinical murine models used in our study offer compelling evidence that reducing macrophage abundance is beneficial for improving the treatment outcome of HDACis. This effect is relatively independent of the adaptive immune responses, as it was observed in both immunocompetent mice and nude mice to a similar extent. Apart from preventing macrophage phagocytosis, the elevated CD47 expression in tumor cells also reciprocally increased the M2-like phenotype of macrophages. This effect is CD47-dependent as it was abolished by CD47 neutralization. Actually, SIRPα expression is not restricted to macrophages. The CD47 ligation-induced SIRPα signaling has been documented to be crucial for the development of dendritic cells [14, 15]. On the other hand, CD47 is also expressed in various hematopoietic and non-hematopoietic cells. Therefore, CD47 upregulation induced by HDACis may influence the cross-regulation among other types of cells. In addition to their application in cancer treatment, HDACis are also effective in managing a range of diseases, such as colitis [27], rheumatoid arthritis (RA) [28], and Duchenne muscular dystrophy (DMD) [29]. How the HDACi-induced CD47 influences the therapeutic outcome of these diseases needs further investigation.

The most distinctive microenvironmental feature of the colon is the inhabited microbiota and the metabolites they produce [30]. Our previous study reported that CRC patients exhibited remarkably reduced fecal SCFA contents compared to healthy subjects [8], presumably due to the decreased abundance of SCFA-producing bacteria. Here, we discovered positive correlations between tumor CD47 expression and fecal SCFA contents, particularly butyrate. This finding implies that CRC patients with high SCFA production may be more likely to benefit from CD47-SIRPα blockade immunotherapy. Under clinical settings, butyrate supplementation can be achieved through various approaches, such as fecal microbiota transplantation (FMT), butyrate capsules, or butyrate-producing probiotics [31, 32]. Thus, the potential, macrophage-mediated immunosuppression should be considered for CRC patients who received butyrate supplementation.

Due to their extreme functional plasticity, the “double-edged” nature of macrophages in tumor progression has been well-recognized for a long time. Here, we identified CD47 as a novel determinant that can tell us whether macrophages act as a friend or foe in the TME. This finding is supported by clinical evidence showing that macrophages exhibit opposite prognostic significance in CD47^hi^ and CD47^low^ tumors, both in CRC patients and breast cancer patients. Hence, our study suggests at least two potential implications: (1) Patients with higher TAM abundance may have higher possibility of benefiting from CD47-SIRPα blockade immunotherapy. (2) For patients with high CD47 expression, therapies aimed at eliminating macrophages may be more efficacious.

## Conclusions

In summary, the present study underscores that targeting macrophage infiltration or blocking CD47-SIRPα axis is potentially necessary for reversing the resistance to HDACi therapies.

## Supplementary Information

Supplementary Material 1

## Data Availability

Data is provided within the manuscript or supplementary information files.
