# Supplementary material for: CD47 blockade reverses resistance to HDAC inhibitor by liberating anti-tumor capacity of macrophages
Source: J Exp Clin Cancer Res. 2025 Feb 24;44:67. doi: 10.1186/s13046-025-03335-5 (PMC11849317; doi:10.1186/s13046-025-03335-5)
Supplement: Supplementary file 1 — Supplementary Material 1 [file 13046_2025_3335_MOESM1_ESM.pdf]

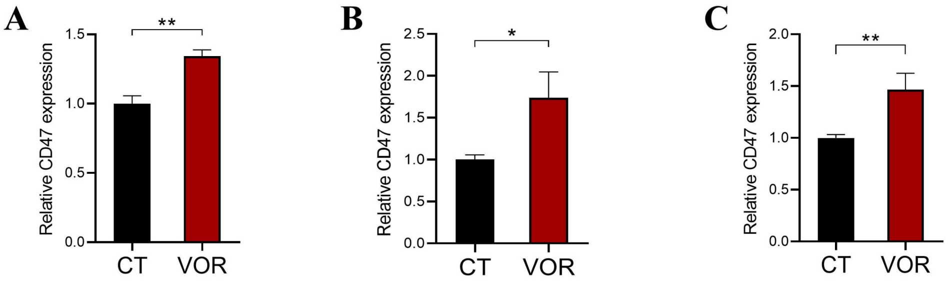

Figure S1. SW480 CRC cells (A), PANC-1 pancreatic cancer cells (B), and MDA-MB-231 breast cancer cells (C) were stimulated with VOR for 12 hours, the expression of CD47 was evaluated by QPCR. *p < 0.05; **p < 0.01; unpaired, two-tailed Student's t test.

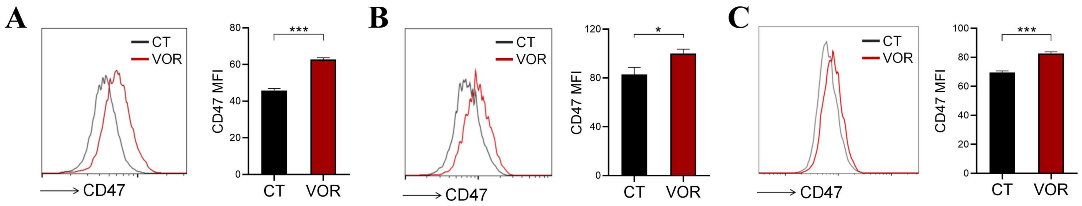

Figure S2. SW480 CRC cells (A), PANC-1 pancreatic cancer cells (B), and MDA-MB-231 breast cancer cells (C) were stimulated with VOR for 24 hours, the expression of CD47 was evaluated by flow cytometry. *p < 0.05; ***p < 0.001; unpaired, two-tailed Student's t test.

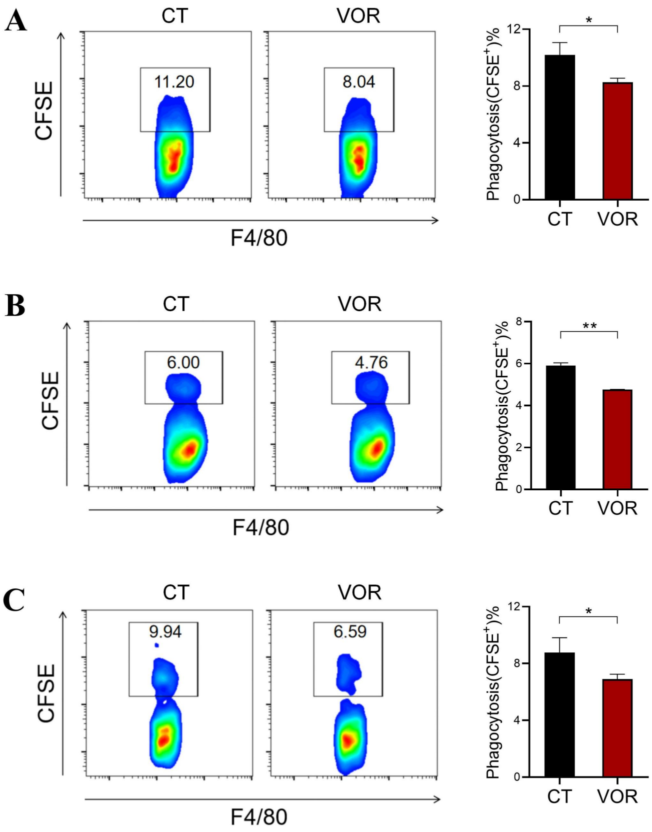

Figure S3. SW480 CRC cells (A), PANC-1 pancreatic cancer cells (B), and MDA-MB-231 breast cancer cells (C) were treated with VOR for 24 hours and labeled with CFSE, followed by the co-incubation with mouse peritoneal macrophages overnight. The phagocytosis of macrophages was evaluated by flow cytometry. $*p < 0.05$; $**p < 0.01$; unpaired, two-tailed Student's t test.

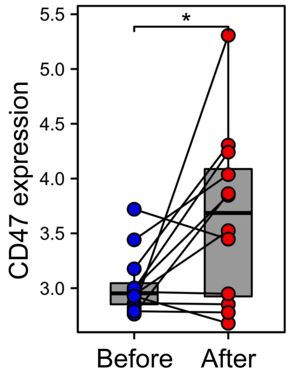

Figure S4. The expression of CD47 in isolated $CD34^+$ cells from patients pre- and post-entinostat treatment was analyzed using a GEO dataset (GSE16625). $*p < 0.05$, paired,

two-tailed Student's t test.
